# On optimization of Spin-Forming Process Parameters for Magnesium Alloy Wheel Hub Based on Gray Relational Analysis

**DOI:** 10.3390/ma17040959

**Published:** 2024-02-19

**Authors:** Zheng Zhang, Yongting Lan, Haochuan Ding, Yuanhang Xie

**Affiliations:** College of Mechanical and Automotive Engineering, Guangxi University of Science and Technology, Liuzhou 545616, China; zhng_ae@163.com (Z.Z.); 15690863618@163.com (H.D.); x15536303134@163.com (Y.X.)

**Keywords:** magnesium alloy wheel hub, spin-forming, forming quality, gray relational degree

## Abstract

To study the influencing factors of process parameters on the wall thickness deviation and internal warpage deviation of the workpiece in magnesium alloy wheel hub spin molding, a two-pass heterogeneous spin molding model is proposed. To ensure the accuracy of the simulation results, the stress–strain data of AZ31 magnesium alloy at different temperatures and different strain rates were obtained through tests. Wall thickness deviation and internal warp deviation after molding were used as evaluation indexes of workpiece molding quality. ABAQUS software facilitated the numerical simulation and analysis of the magnesium alloy wheel hub spinning process. Gray relational degree analysis optimized the first-pass process parameters, elucidating the impact of the axial offset, the thinning ratio, and the feed ratio on forming quality. The application of optimized parameters in the hub spinning simulation resulted in a substantial 28.84% reduction in wall thickness deviation and a 4.88% reduction in inner diameter deviation. This study underscores the efficacy of employing Gray Relational Analysis for comprehensive parameter optimization, ensuring wheel hub quality. Moreover, it provides a theoretical foundation for enterprises to expedite research and development cycles and minimize associated costs.

## 1. Introduction

Automobile wheels shoulder the vehicular load and perform critical functions, including power transmission, shock absorption, and noise reduction. The inherent strength, stiffness, and overall performance of these wheels plays a pivotal role in determining the safety and driving comfort of the vehicle [1]. With the release of “Made in China 2025”, the development goals for the energy conservation and emission reduction in the automotive industry have become clearer [2]. This further promotes the alloying and light weight of car wheels. Magnesium alloy, regarded as an environmentally friendly material in the 21st century, exhibits outstanding shock absorption, high specific strength, and low density. It finds extensive applications in aerospace, automotive, and 3C fields, establishing itself as an excellent lightweight material [3,4,5].

The spinning process can refine metal grains and create fibrous structures, thereby enhancing the mechanical properties of materials [6]. Currently, numerous scholars employ numerical simulation methods to investigate the impact of process parameters on the spinning forming process. Li et al. [7] conducted a study on the variation in wall thickness deviation and spin pressure under different process parameters using the numerical simulation method. They optimized the process parameter combinations for multi-pass large-diameter-to-thickness-ratio cylindrical parts and proposed a 10-pass spinning forming process. H. Lexian et al. [8] conducted research on the shape of the rollers by establishing a finite element model and code. By improving the shape of the rollers to form the dome of cylindrical parts, a better forming quality can be achieved. M.S. Mohebbi et al. [9] conducted research on the single roller forming of cylindrical parts through a combination of finite element simulation and experimental methods. They revealed the distribution of various strains during the spinning process and the conclusion that frictional work can be ignored. K. Essa et al. [10] conducted research on sheet metal spinning forming into cylindrical parts through finite element simulation. Through two sets of DOE experiments, the optimal combination of process parameters and the impact mechanism of important process parameters on forming quality were determined. The results showed that the optimized product quality improved by 22%. Liu et al. [11] used numerical simulation to investigate the deformation patterns and factors affecting the forming quality of thin-walled tapered parts with inhomogeneous cross-sections during hot spinning. Xia et al. [12] investigated the equivalent stress distribution of GH1140 alloy in the spinning process and the effect of different process parameters on the spinning force magnitude by numerical simulation. The residual stress distribution after unloading and the causes of defects were also analyzed by experimental comparison. Wang et al. [13] employed numerical simulation in conjunction with a one-factor test to examine the influence of various process parameters on the residual stress of connecting rod bushings during the spinning process.

Of course, there are also scholars that have carried out research on the metal flow and the rebound phenomenon in the spinning process. Mohammad Sadegh Mohebbi et al. [14] conducted research on the spinning forming of AZ31 magnesium alloy cylindrical parts through a combination of simulation and experimentation. The minimum spinning temperature for AZ31 magnesium alloy should be above 522 K, and the material state before and after spinning and annealing should be compared. It is recommended that scholars use a linear feed method to set the motion trajectory of the roller. M. Jahazi et al. [15] investigated the effects of different process parameters and heat treatment processes on the spinning forming of D6ac steel; explored the effects of different process parameters on defects such as surface ripples, cracks, and voids in materials; and obtained the optimal combination of forming parameters by controlling heat treatment parameters. Hamid R et al. [16] conducted spinning tests on cylindrical parts to investigate the effects of different thinning rates on the surface forming quality of the workpiece, as well as the microstructure and mechanical properties of the material. M. Keneshlou et al. [17] conducted a combined numerical simulation and experimental study on the spinning process of non-circular workpieces to unveil the rebound and strain distribution of deformed parts. The findings indicated that the shell unit is more effective for rebound prediction, whereas the solid unit excels in strain prediction. There are also some scholars that have explored at the microscopic level the mechanism of spinning forming and the improvement in the spinning process. Xia et al. [18] introduced a novel approach that integrates a three-dimensional thermal processing map with numerical simulation to address issues related to underfilling and depression at the back of ribs in the spinning process of magnesium alloy thin-walled cylindrical parts with internal ribs. This study unveiled the forming mechanisms underlying different defects, analyzed the metal flow and stress–strain state of distinct workpiece regions, and assessed the power consumption efficiency in the spinning process. This comprehensive analysis aimed to determine the appropriate processing window for AK61 magnesium alloy. A. A. Abd-Eltwab et al. [19] conducted research on the ball spinning forming of cylindrical parts with inner ribs through a combination of theory and experiment. The conclusion was drawn that the spindle speed and feed rate have a significant impact on the forming quality, and the spinning force during the forming process was analyzed. M. Watson H. et al. [20] investigated the effects of six parameters, including feed rate, workpiece thickness, and roller speed, on forming quality by designing 47 sets of experiments, and they analyzed the wrinkling mechanism caused by the residual bending moment and the influence of Young’s modulus on residual torque.

Due to the dense hexagonal crystal structure of magnesium alloys, they cannot meet the number of slip systems required for the uniform deformation of polycrystalline materials at room temperature, making it difficult to perform plastic processing at room temperature [21,22]. The wheel spin molding process is a complex nonlinear process and involves the complex coupling of multiple process parameters and roller trajectories. The selection of process parameters should be adjusted based on the wheel hub model and different processing passes, and it is difficult to obtain the optimal combination of process parameters in the experiment. Therefore, this article proposes a two-pass heterogeneous spin-forming model based on ABAQUS2021 finite element software to study the influence of different process parameter combinations on spinning forming quality. The analysis delves into changes in the stress, strain, and metal flow during the wheel hub spinning process, combining the Gray Relational Analysis method to comprehensively select the optimal combination of process parameters and providing a theoretical basis for enterprises to shorten the research and development cycle and reduce research and development costs.

## 2. Finite Element Model of Spinning Forming

### 2.1. Establishment of Geometric Models

**Figure 1:**
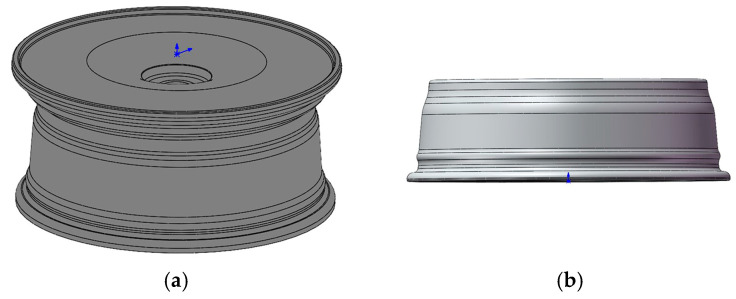
Model of wheel hub: (**a**) designed standard wheels; (**b**) simplified wheel model.

Due to the change in the internal structure of the material during the spinning process, the volume changes. Based on established experience, a volume ratio of (1.03 ± 0.1):1 was deemed more suitable for the workpiece before and after spinning. The 17 × 7.5 J standard hub was designed, as shown in Figure 1a. Since the wheel spoke part material does not undergo spinning forming in the wheel rim process, a simplification was applied to expedite calculations. Only the section of the wheel rim participating in the spinning forming was considered, with the ABAQUS software utilizing the “TIE” constraint to effectively substitute the influence of the wheel spoke part on the model. The resulting simplified wheel model is illustrated in Figure 1b.

**Figure 2:**
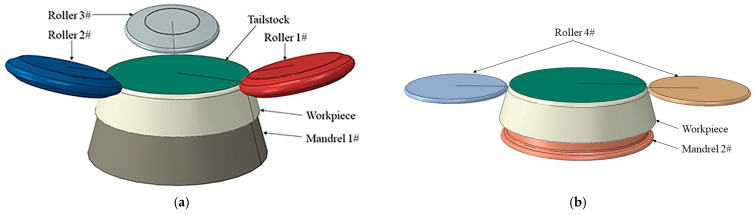
Two-pass spinning model: (**a**) first pass model; (**b**) second pass model.

To achieve precise control of the forming quality of the workpiece during the entire spinning process, two-pass spinning was used, as shown in Figure 2. Each pass model comprises three components: rollers, workpiece, and molds. The first pass involves a strong spinning with staggered distance for three rollers, primarily focusing on thinning and elongating the workpiece. This step significantly influences the final molding quality of the wheel. This article focuses on optimizing and analyzing the one-pass forming process in the wheel hub spinning process. The second pass is a conventional spinning pass for shaping the wheel, mainly accomplishing the overall molding of the workpiece. The workpiece type was a 3D deformable solid, the rollers were a 3D analytical rigid shell, and the mandrels were a 3D discrete rigid shell.

### 2.2. Material Properties

The rollers and mandrels were modeled as rigid bodies, while the workpiece was composed of AZ31 magnesium alloy. The specific composition is detailed in Table 1. The material’s density was 1780 kg/m³, with a Poisson’s Ratio of 0.34 and a Young’s modulus of 45 GPa. The equivalent strain rate $\dot{\varepsilon }$ during the spinning process can be calculated by the following equation [23,24]:

(1) $$\dot{\varepsilon }=\frac{2}{\sqrt{3}t_{0}}\frac{v_{i}sin\alpha_{i}}{\left(1-\varphi_{t}\right)}$$

where $t_{0}$ is the initial workpiece thickness; $v_{i}$ is the metal flow velocity in front of the rollers; $\alpha_{i}$ is the forming angle of the rollers; and $\varphi_{t}$ is the pass thinning ratio.

**Figure 3:**
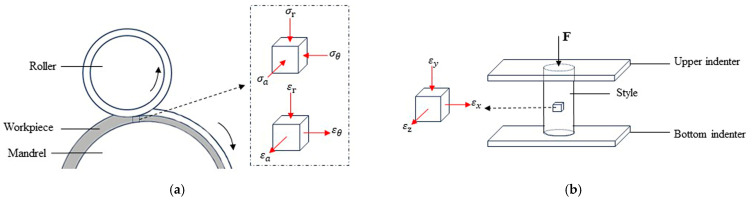
Comparison of strain states: (**a**) strain state during spinning process; (**b**) strain state during uniaxial compression process.

**Figure 4:**
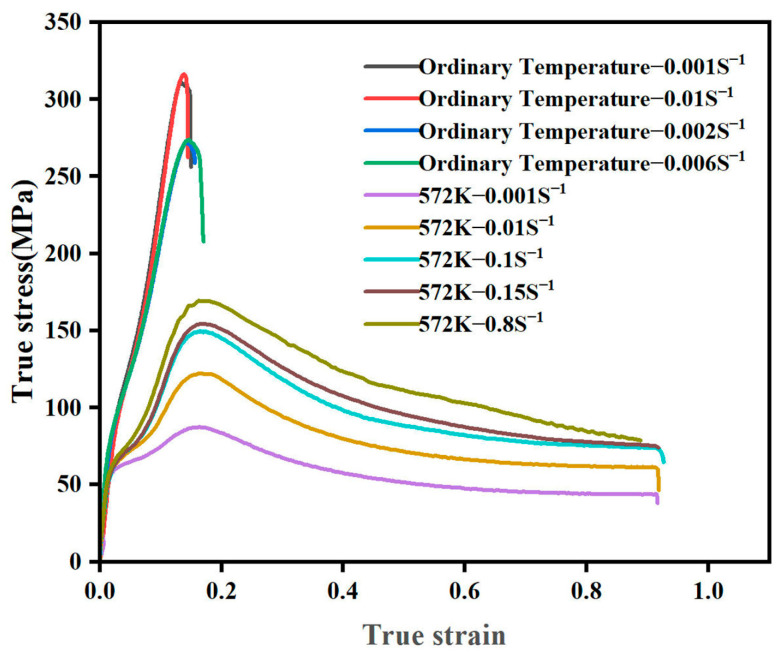
True stress–strain curves of AZ31 magnesium alloy in compression at different temperatures and strain rates.

As shown in Figure 3, the material strain state during the spinning process is similar to that of the unidirectional compression test. During spinning, the material is subjected to radial, axial, and circumferential compressive stresses, including radial compressive strain, axial tensile strain, and tangential tensile strain [25,26]. Therefore, the stress–strain data measured from the compression test were used as the material model. In order to ensure the accuracy of the simulation, this paper combined with Equation (1) measures the real stress–strain curves of AZ31 magnesium alloy at different temperatures and strain rates using the compression test, as shown in Figure 4, where the yield strength and ultimate strength at a temperature of 572 K and different strain rates are shown in Table 2. The stress–strain data after the yield point of the compression curve were selected and are defined in the “Materials” module of the ABAQUS2021 software.

### 2.3. Contact Properties and Meshing

The optimum hot working range of AZ31 magnesium alloy is 572–672 K [27]. In the spinning process, the workpiece rotates continuously with the spindle. A temperature that is excessively high can soften the material, leading to defects such as burrs. Consequently, a processing temperature of 572 K was selected to both minimize time consumption and maintain the forming quality of the workpiece.

The coefficient of friction between the workpiece and the roller was set at 0.15, while the coefficient of friction between the workpiece and the mandrel was established at 0.02, utilizing the penalized contact algorithm [28]. The contact between the workpiece and the mandrels, as well as the tailstock, was modeled using a “TIE” method, simulating the rigid effect of the wheel spoke and mold on the reverse flow of the workpiece material.

The workpiece was a three-dimensional deformable solid model, and the neutral axis algorithm was used to divide it into hexahedral meshes, with the element type being C3D8R. This type of unit can provide a more detailed characterization of structural deformation in the analysis of complex structures. The number of units divided was 41181, and the number of nodes was 55650. Mandrel 1# and mandrel 2# were three-dimensional discrete rigid body models, and the neutral axis algorithm was still used to divide them into hexahedral meshes. However, its cell type was R3D4, the number of cells divided were 5506 and 3677, and the numbers of nodes were 5643 and 3840, respectively.

### 2.4. Boundary Conditions

Due to the “TIE” connection settings made in the contact section, it was only necessary to set the rotational speed around the spindle at the RP point of the mandrel and limit the remaining 5 degrees of freedom.

For the first pass, the three rollers were staggered to move in the direction of mandrel-1#’s busbar. The speed of movement of the reference point of the rollers in its local coordinate system was set so that the three rollers can complete the axial and radial feed. At the same time, it releases its rotational degrees of freedom around its own local coordinates, restoring its rotation phenomenon caused by friction.

In the second pass, the outline of the rim bus deviates from the wall thickness and was represented in ABAQUS using the 3D-Deformable-Wire method. A finer mesh was delineated for it, and the INP file was exported. By subtracting the initial point coordinates from the grid node coordinates and organizing them, the motion trajectory of the rollers in the local coordinate system was obtained. A displacement boundary condition was applied to the second pass rollers to move along a complex rim profile. The rotational freedom of the rollers was released around its own local coordinates, restoring its self-rotation phenomenon caused by friction. With this method, any complex motion trajectory can be defined in ABAQUS.

### 2.5. Verification of Finite Element Model

**Figure 5:**
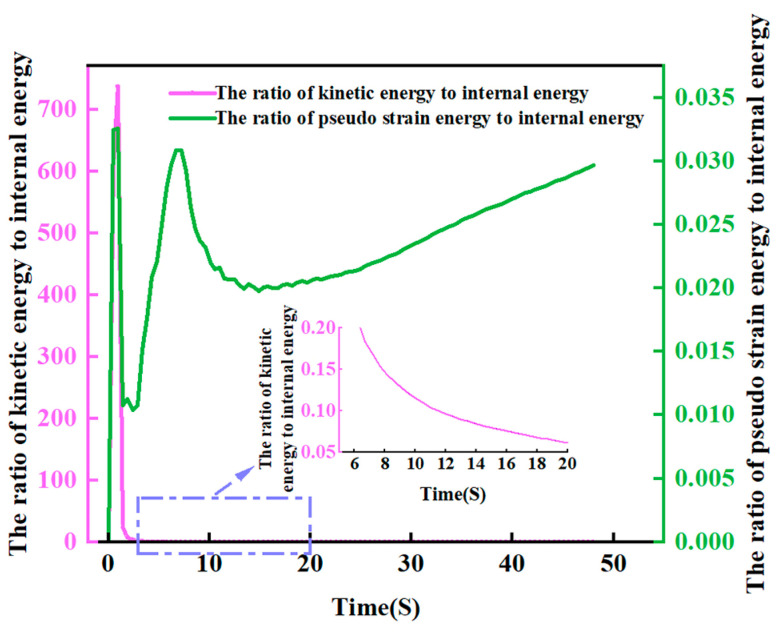
The ratio of kinetic energy to internal energy and the ratio of pseudo-strain energy to internal energy vary with time.

The analysis employed a dynamic explicit approach (ABAQUS/Explicit), with the analysis step time set to match the actual spinning duration. To enhance computational efficiency, mass scaling techniques were applied, but careful consideration is essential when defining the mass scaling factor. Typically, when the ratios of kinetic energy to internal energy and pseudo-strain energy to internal energy remain below 10% for the majority of the time, it indicates that the mass scaling factor and the hourglass effect of the subdivided grid are within reasonable limits [29]. The variation curves of these ratios with a mass scaling factor of 1200 are depicted in Figure 5.

As depicted in Figure 5, the ratio of kinetic energy to pseudo-strain energy experiences a sharp increase during the contact among the rollers and the workpiece, subsequently decreasing to less than 5 percent. Meanwhile, the ratio of pseudo-strain energy to internal energy initially exhibits fluctuations in the curve, attributed to mesh variations at the onset of roller–workpiece contact. However, it stabilizes and remains below 3% for the majority of the simulation time. This analysis underscores that the impact of mass scaling and mesh hourglasses on the simulation results is within acceptable limits, affirming the validity of the finite element model.

### 2.6. Stress–Strain Analysis of Simulation Results

**Figure 6:**
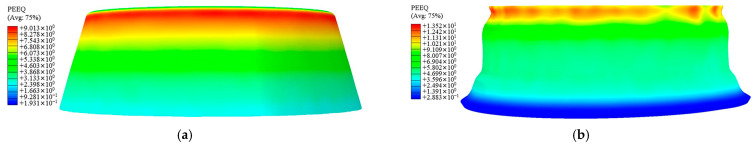
Two-pass sub-equivalent plastic strain distribution: (**a**) equivalent strain distribution in the first pass; (**b**) equivalent strain distribution in the second pass.

A feed ratio of 1.2 mm/r; three rollers’ thinning ratios of 15%, 23.53%, and 28.57%; and an axial offset of 12 mm were selected for the analysis. The corresponding compression curve in Figure 3 is at 572 K and 0.1 s^−1^. As shown in Figure 6a, the equivalent strain on the outer surface undergoes a process of increasing and then decreasing during the first pass of workpiece deformation. A “strain-ring” region of high strain appears in the upper center of the workpiece. Its reason was investigated, firstly, because at the beginning of the spinning, by the influence of the “rigid-end” of the flange in the undeformed area, the inner cone angle of the workpiece will be relatively large, resulting in an increase in the rate of wall thickness reduction. In the middle and late stages of the spinning process, as the influence of the flange becomes smaller, the change in the thinning ratio due to the increase in the inner cone angle decreases accordingly. Secondly, most of the metal flows to the back of the rollers during the spinning process, but there will still be a small amount of metal flowing to the front of the rollers to produce metal buildup. This results in an increase in the actual thinning ratio of the rollers, which leads to an increase in the corresponding equivalent strain. As the spinning process progresses, all three spinning wheels come into contact with the workpiece. At this point, the influence of the “rigid end” of the flange on the workpiece is gradually reduced, so the bulge is reduced and the deformation of the workpiece gradually returns to normal.

As depicted in Figure 6b, during the second pass of workpiece deformation, the equivalent strain on the external surface exhibits a pattern of increase followed by a decrease. In the upper center of the workpiece, there exists a “strain-ring” area with high strain, while the lower part retains a certain amount of machining allowance. This is attributed to the significant grooves in the upper part of the mandrel at the initiation of the second pass, which needs to be filled during the workpiece forming, leading to substantial strains. As the radius of the first pass is larger as it approaches the lower part of the hub, the second pass can be laminated without a larger filler, so the strain undergoes an increasing and then decreasing trend.

**Figure 7:**
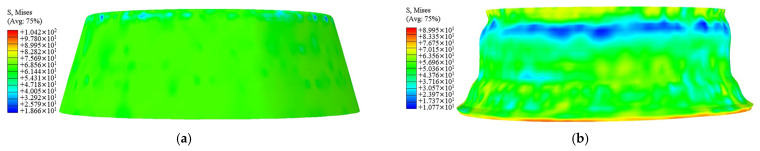
Two-pass sub-equivalent stress distribution: (**a**) equivalent stress distribution in the first pass; (**b**) equivalent stress distribution in the second pass.

As shown in Figure 7a, the stress distribution is relatively uniform and the surface forming quality is good during the first pass of spinning. Throughout the spinning process, stress maxima occur in the working area of the rollers as well as at the top of the workpiece. In Figure 7b, during the second spinning and forming process, the rim angle undergoes considerable changes. In this stage, the equivalent force of the workpiece is primarily distributed in the contact area among the rollers and the workpiece, as well as in the top and lower areas of the workpiece. The maximum stress of the first pass occurs in the section where the workpiece is “TIE” to the mold, reaching a peak value of 102.1 MPa. The maximum value of the second-pass secondary stress appears in the convex peak of the workpiece, reaching a maximum of 89.95 MPa. Notably, the maximum value from the two passes does not exceed the ultimate strength of the material, which is 149.44 MPa.

This is mainly due to two reasons. Firstly, during the forming process, the top of the workpiece is “TIE” to the mandrel, mimicking the effect of the wheel spoke portion on the rim. At this time, the mandrel drives the entire workpiece to rotate, i.e., the stress applied to the workpiece by the mandrel. The second factor is the cumulative impact of the shear force from the rollers on the bottom of the workpiece during the loading process.

## 3. Experimental Design and Evaluation Indicators

### 3.1. Sampling Locations and Evaluation Indicators

During the spinning process of the wheel hub, the rollers are subjected to point-by-point plastic deformation, and the deformation process is affected by multiple factors. The wall thickness of the workpiece is prone to deviation, and localized thinning phenomena can cause the weakening of performance, which in turn creates areas of quality risk. The wall thickness deviation is defined as the variance between the actual and theoretical wall thickness in the thickness direction of the workpiece. The internal warp deviation, on the other hand, measures the distance between the inner surface of the workpiece and the mandrel after spinning, which can characterize the degree of hub expansion or lamination.

**Figure 8:**
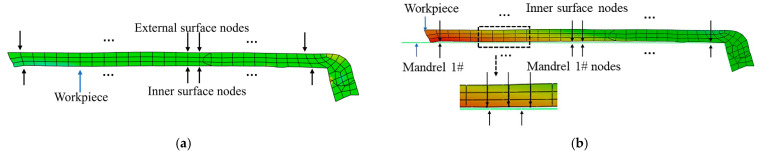
Schematic diagram of sampling nodes for each evaluation indicator: (**a**) sampling points for wall thickness deviation; (**b**) sampling points for internal warp deviation.

**Figure 9:**
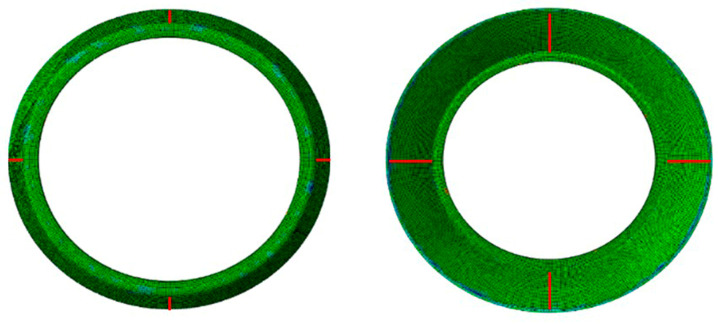
Schematic diagram of sampling location.

As depicted in Figure 8, the wall thickness deviation of the workpiece is determined by calculating the difference between the node coordinates of the outer surface and the inner surface. Similarly, the internal warp deviation is obtained by assessing the difference between the nodal coordinates of the inner surface and those of the mandrel. As shown in Figure 9, four positions are taken at each of the positions corresponding to the inner and outer surfaces of the workpiece after spinning, and all the nodes on their paths are selected at each position to extract their node coordinates. The internal warp deviation and wall thickness deviation are expressed by finding the standard deviation of the post-processed coordinate changes.

### 3.2. Design of Orthogonal Experimental Table

The feed ratio is the distance the rollers move straight down the workpiece bus for each revolution of the workpiece. The thinning ratio is the ratio of the amount of change in the thickness of the workpiece during the movement of the rollers to the initial thickness. The axial offset is the distance that each roller is staggered in the direction of the workpiece busbar. These three process parameters were chosen as the factors for the experiment. Due to the role of all three rollers in reducing the thickness during the first pass, the reduction ratio of the roller is expressed as a group number in the experiment, as shown in Table 3. Wall thickness deviation and internal warp deviation were selected as evaluation indexes, and a three-factor, four-level orthogonal experimental program was designed. The orthogonal experimental program and results are shown in Table 4.

### 3.3. Verification of Cylindrical Component Testing

**Figure 10:**
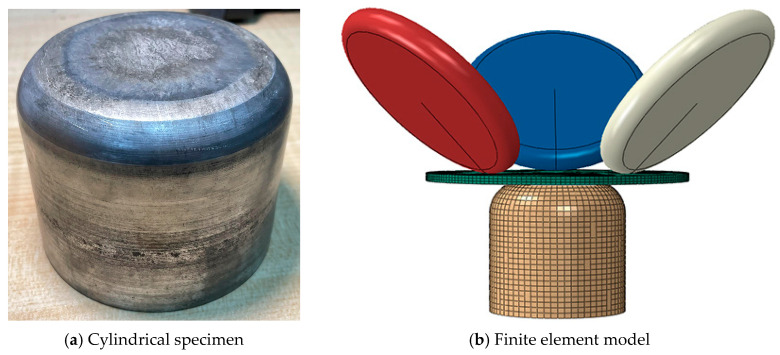
Verification model for cylindrical components.

According to the research conclusions of existing scholars, the spinning results of cylindrical parts can be successfully applied to wheel hub spinning, and the two can be mutually confirmed, but the process parameters for wheel hub spinning need to be more stringent [30]. Therefore, to ensure the effectiveness of the established wheel hub spinning model, simulation and experimental verification were conducted through cylindrical component spinning. When establishing the cylindrical part model, the mesh division method, contact attribute settings, and boundary conditions are consistent with the aforementioned hub model. The spinning style and model of cylindrical parts are shown in Figure 10.

The results of the cylindrical part spinning test and simulation are shown in Table 5, where the error of the wall thickness deviation between the two is about 7.72%, and the error of the internal warp deviation is 6.92%. There is good consistency between the experimental and simulation values of cylindrical components, indicating that the modeling method is reasonable and effective.

## 4. Result Analysis and Optimization

When employing Gray Relational Analysis for multi-objective optimization, the problem can be reformulated into a single objective for comprehensive analysis. This method effectively characterizes the magnitude and order of influence between factors. By calculating the gray relational coefficient and gray relational degree for each evaluation index, the average gray relational degree of each index can be determined. This approach enables the assessment of the sensitivity of each process parameter to the evaluation index and facilitates the identification of the optimal combination of process parameters through optimization. The following Gray Relational Analysis is carried out based on the 16 sets of wall thickness deviation and internal warp deviation results from the orthogonal tests in Table 4 as optimization data.

### 4.1. Gray Relational Calculation and Results

The wall thickness deviation and internal warp deviation should be as small as possible. Therefore, the lower-the-better formula was used to discretize the data [31].

(2) $$x_{i}\left(k\right)=\frac{\max x_{i}^{0}\left(k\right)-x_{i}^{0}\left(k\right)}{\max x_{i}^{0}\left(k\right)-\min x_{i}^{0}\left(k\right)}$$

where $x_{i}\left(k\right)$ is the processed test data; $x_{i}^{0}\left(k\right)$ is the unprocessed test data; i=1, 2, …, n; n is the number of tests; k=1, 2, …, m; m is the number of test groups; $\max x_{i}^{0}\left(k\right)$ is the maximum value of unprocessed data; and $\min x_{i}^{0}\left(k\right)$ is the minimum value of unprocessed data.

The gray relational coefficient $\xi_{i}\left(k\right)$ is calculated as [32]

(3) $$\xi_{i}\left(k\right)=\frac{\Delta_{\min}+\varphi \Delta_{\max}}{\Delta_{0i}\left(k\right)+\varphi \Delta_{\max}}$$

(4) $$\Delta_{0i}\left(k\right)=\left|X^{0}\left(k\right)-x_{i}\left(k\right)\right|$$

where $\Delta_{\min}$ is the minimum value of deviation, $\Delta_{\max}$ is the maximum value of deviation, $\Delta_{0i}\left(k\right)$ is the deviation between the reference target $X^{0}\left(k\right)$ and the comparison target $x_{i}\left(k\right)$, and φ is the deviation coefficient. In this paper, φ=0.5 and $X^{0}\left(k\right)=1$ [33,34].

The gray relational degree $\gamma_{i}$ of each group of experiments is calculated. The calculation formula is as follows [35]:

(5) $$\gamma_{i}=\frac{1}{n}\sum_{i=1}^{n}\xi_{i}\left(k\right)$$

The gray relational coefficients for the wall thickness deviation and internal warp deviation, along with the gray relational degree of each group, were obtained from the data in Table 3 and calculated using the aforementioned formulas, as presented in Table 6.

As observed in Table 6, among the 16 sets of tests, the 10th set of process parameter combinations exhibited the highest gray relational degree of 0.85. Consequently, the process parameters of Group 10 represent the optimal combination that simultaneously minimizes both wall thickness deviation and inner diameter deviation within the 16 experimental groups. Specifically, this optimal combination includes a feed ratio of 1.2 mm/r; three rollers with thinning ratios of 15%, 23.53%, and 28.57%; and an axial offset of 12 mm.

The average gray relational degree is obtained by calculating the average of the gray relational degree for the same values for different process parameters in Table 6. A higher average gray relational degree indicates a more substantial influence of the corresponding values of the process parameters on the evaluation indicators.

Table 7 presents the average gray relational degree for different values of each factor. The order of influence of various values on forming quality under each factor is as follows: feed ratio (1.4 mm/r > 1.2 mm/r > 1 mm/r > 0.8 mm/r), thinning ratio group number (3 > 2 > 1 > 4), and axial offset (12 mm > 10 mm > 6 mm > 8 mm). Since the maximum average gray relational degree of different values under each factor is the value of the best process parameter, the best combination of process parameters obtained by the Gray Relational Analysis method is a feed ratio of 1.4 mm/r, thinning ratio group number of 3, and an axial offset of 12 mm.

Based on the average gray relational degree under different values for each factor, the extreme difference values between the four values under each factor were calculated. The order of influence of each factor on forming quality was explored through extreme difference analysis. As observed in Table 6, the axial offset exhibits the largest extreme different value at all levels, indicating the greatest impact on the quality of wheel molding. The degree of influence for each factor is ranked as follows: the axial offset, the thinning ratio, and the feed ratio.

### 4.2. Optimization and Validation of Results

The more optimal combination of process parameters (the feed ratio of 1.4 mm/r; the three rollers’ thinning ratios of 15%, 17.65%, and 28.57%; and the axial offset of 12 mm) optimized using Gray Relational Analysis was not included among the 16 sets of data from the orthogonal tests. Therefore, the model parameters were adjusted for simulation verification, and the test results were compared with those of group 10 in Table 4, as shown in Table 8.

After optimization using the Gray Relational Analysis method, the wheel wall thickness deviation is reduced by 28.84%, and the internal warp deviation is reduced by 4.88% compared to the results of group 10. It is evident that obtaining the optimal process parameter combination for the first pass through the Gray Relational Analysis method is feasible. Under improved process parameters, the quality of the workpiece obtained is higher. The simulation results of optimizing the workpiece are analyzed as follows.

**Figure 11:**
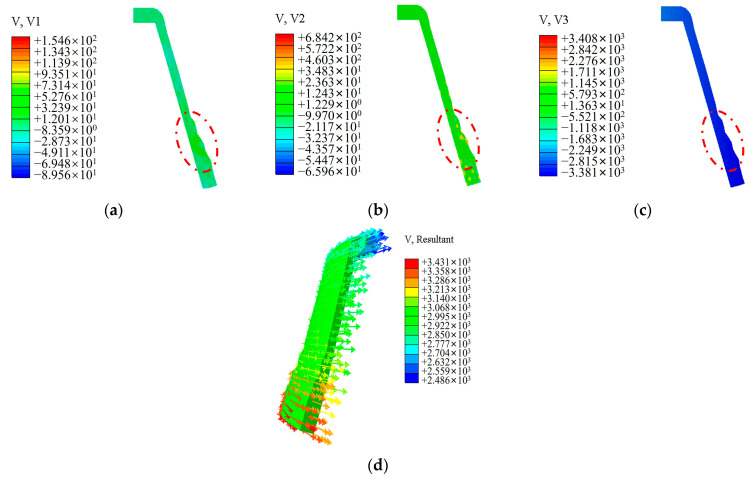
Flow of material in the deformation zone during spinning: (**a**) cloud map of axial flow rate of material; (**b**) cloud map of radial flow rate of material; (**c**) cloud map of tangential flow rate of material; (**d**) flow direction of materials.

Figure 11 illustrates the metal flow in the deformation zone during spinning. The figure reveals that the metal flow velocity on the outer surface of the workpiece in direct contact with the rollers is the highest, gradually decreasing from the outside to the inside along the wall thickness direction. The material flows radially along the wall thickness direction, moving from the outside to the inside. In the axial direction, the metal flow aligns with the axial feed direction of the rollers. In the circumferential direction, the metal flow is influenced by the rotation of the workpiece, resulting in a direction opposite to the rotation. In the undeformed region, metal flows horizontally along the axial direction, facilitating axial elongation. In the deformation zone, metal moves toward the mandrel under the rollers’ action, leading to continuous thinning and adherence to the mandrel. This process condition effectively prevents metal accumulation in front of the rollers in the deformation zone.

**Figure 12:**
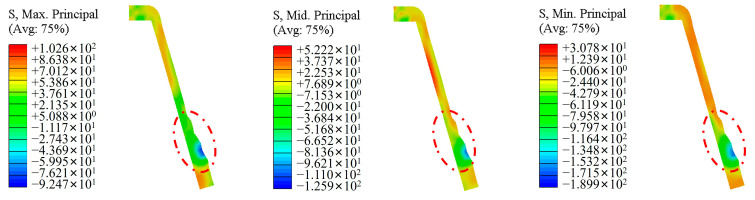
The cloud diagram of the principal stress distribution during the spinning process.

Figure 12 shows the distribution of principal stresses in the deformation zone during spinning. Due to the combined action of the rollers and the circumferential metal on the outer surface of the workpiece, the three principal stresses on the outer surface are all relatively large, while the principal stress on the inner surface is relatively small. In the action zone of the rollers, the three principal stresses are all compressive stresses. Therefore, the deformation zone is under a three-dimensional compressive stress state, and the magnitude of the compressive stress gradually decreases from the outer to the inner surface along the wall thickness direction, which is consistent with the theoretical model during spinning [18].

**Figure 13:**
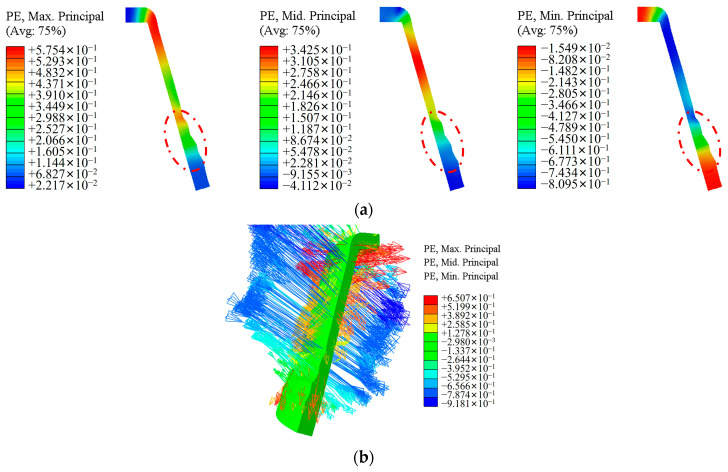
Distribution law of principal strain during spinning process: (**a**) the cloud diagram of the principal stain distribution during the spinning process; (**b**) vector diagram of principal strain.

Figure 13 shows the cloud diagram of the distribution law of the principal strain during the spinning process. As shown in Figure 13a, the metal material of the workpiece experiences tensile strain in both the axial and circumferential directions, and compressive strain in the radial direction, which is consistent with the theoretical model during spinning. Figure 13b shows the directions of the three principal strains. The red arrow represents the first principal strain direction, which is parallel to the surface of the workpiece and along the workpiece busbar for most of its position except for the upper corners. It indicates that the metal material of the workpiece undergoes tensile strain along the direction of the busbar, and the workpiece is elongated. The blue arrow represents the direction of the minimum principal strain, which is perpendicular to the surface of the workpiece and along the thickness direction of the workpiece. This indicates that the metal material of the workpiece has undergone compressive strain and has been thinned along the wall thickness direction. The yellow arrow is the direction of the second principal strain, indicated as the axial strain of the workpiece, which has a relatively small value. The above analysis shows that during the first pass of hub forming, the workpiece relies on thinning in the wall thickness direction to achieve axial elongation.

## 5. Conclusions

Based on the experimentally measured data of the compression process of AZ31 magnesium alloy and the finite element software ABAQUS, a numerical model of the two-pass heterogeneous spinning of magnesium alloy wheels has been established to produce high-quality wheel products. The main findings of this study are summarized as follows:

1. A model for the two-pass molding of hub spinning is proposed. The first pass is a three-roller staggered strong spinning pass, and the second pass is a pair of general spinning shaping passes. Two-pass processing can obtain a better combination of process parameters for each pass and ensure the forming quality of the workpiece for each pass.
2. Gray Relational Analysis is used in the spinning process to demonstrate the feasibility of this method for spinning process optimization. Optimization analysis using Gray Relational Analysis revealed that the axial offset has the most significant impact on the forming accuracy of hub spinning. The order of influence of the three experimental factors on the quality of the initial pass in the hub spinning process is as follows: the axial offset, the thinning ratio, and the feed ratio. The Gray Relational Analysis method identified an optimal process parameter combination with a smaller wall thickness deviation and inner diameter deviation compared to the 10th group of orthogonal experiments, resulting in improved forming quality.
3. Having analyzed in depth the stresses, strains, and metal flow during the spinning of workpieces, it was observed that, during the loading of rollers, the metal in the contact area among the rollers and the workpiece undergoes radial compression and axial/circumferential elongation. Nevertheless, the deformation is constrained by the metal material in the surrounding non-contact area. The metal in the axial front and rear annular regions hinders its circumferential flow.

## Figures and Tables

**Table 1:** Composition of AZ31 magnesium alloy (mass fraction, %).

Element	Al	Zn	Mn	Si	Mg
wt.%	3.0	0.9	0.2	0.3	Ba

**Table 2:** Yield and ultimate strengths of AZ31 magnesium alloy at 572 K and different strain rates.

Temperature (K)	Strain Rate (S^−1^)	Yield Strength (MPa)	Ultimate Strength (MPa)
572	0.001	54.86	87.42
572	0.01	57.71	122.22
572	0.1	60.15	149.44
572	0.15	63.55	154.40
572	0.8	64.90	169.43

**Table 3:** Combination of three rollers’ thinning ratios.

Group Number for Thinning Ratios	Roller 1#	Roller 2#	Roller 3#
1	20%	18.75%	23.08%
2	15%	23.53%	23.08%
3	15%	17.65%	28.57%
4	10%	22.22%	28.57%

**Table 4:** The orthogonal experimental program and results.

Serial Number	Feed Ratio (mm/r)	Group Number for Thinning Ratios	Axial Offset (mm)	Wall Thickness Deviation (mm)	Internal Warp Deviation (mm)
1	0.8	1	6	0.70	0.91
2	0.8	2	8	0.81	0.78
3	0.8	3	10	0.70	0.44
4	0.8	4	12	0.60	0.62
5	1	1	8	1.07	0.49
6	1	2	6	0.55	0.64
7	1	3	12	0.37	0.70
8	1	4	10	0.76	1.10
9	1.2	1	10	0.34	0.67
10	1.2	2	12	0.52	0.41
11	1.2	3	6	0.71	0.62
12	1.2	4	8	1.17	1.43
13	1.4	1	12	0.62	0.43
14	1.4	2	10	0.58	0.51
15	1.4	3	8	0.65	0.54
16	1.4	4	6	0.78	0.45

**Table 5:** Comparison between simulation values and experimental values.

Evaluating Indicator (mm)	Simulation Value	Experimental Value	Error Value (%)
Wall thickness deviation	0.237	0.224	7.72
Internal warp deviation	0.463	0.433	6.92

**Table 6:** Gray relational coefficient and gray relational degree.

Serial Number	Feed Ratio (mm/r)	Group Number for Thinning Ratios	Axial Offset (mm)	Gray Relational Coefficient		Gray Relational Degree
				The Wall Thickness Deviation (mm)	The Internal Warp Deviation (mm)	
1	0.8	1	6	0.54	0.50	0.52
2	0.8	2	8	0.47	0.58	0.52
3	0.8	3	10	0.54	0.94	0.74
4	0.8	4	12	0.61	0.71	0.66
5	1	1	8	0.36	0.86	0.61
6	1	2	6	0.66	0.69	0.68
7	1	3	12	0.93	0.64	0.79
8	1	4	10	0.50	0.43	0.46
9	1.2	1	10	1.00	0.66	0.83
10	1.2	2	12	0.70	1.00	0.85
11	1.2	3	6	0.53	0.71	0.62
12	1.2	4	8	0.33	0.33	0.33
13	1.4	1	12	0.60	0.96	0.78
14	1.4	2	10	0.63	0.84	0.73
15	1.4	3	8	0.57	0.80	0.68
16	1.4	4	6	0.49	0.93	0.71

**Table 7:** Mean gray relational degree at each value of each factor.

Level	Feed Ratio (mm/r)	Group Number for Thinning Ratios	Axial Offset (mm)
1	0.61	0.69	0.63
2	0.63	0.70	0.54
3	0.66	0.71	0.69
4	0.73	0.54	0.77
Range	0.11	0.17	0.23

**Table 8:** Comparison of optimization results.

Sample	Feed Ratio/(mm/r)	Group Number for Thinning Ratios	Axial Offset/mm	Wall Thickness Deviation/mm	Internal Warp Deviation/mm
Group 10	1.2	2	12	0.52	0.41
optimization group	1.4	3	12	0.37	0.39

## Data Availability

The data presented in this study are available on request from the corresponding author.

## References

[1] Dong G., Li S., Ma S., Zhang D., Bi J., Wang J., Starostenkov M.D., Xu Z. (2023). Process optimization of A356 aluminum alloy wheel hub fabricated by low-pressure die casting with simulation and experimental coupling methods. J. Mater. Res. Technol..

[2] Li Q., Zhang Y., Zhang C., Wang X., Chen J. (2022). Analysis Method and Case Study of the Lightweight Design of Automotive Parts and Its Influence on Carbon Emissions. Processes.

[3] Prasad S.V., Prasad S.B., Verma K., Mishra R.K., Kumar V., Singh S. (2022). The role and significance of Magnesium in modern day research—A review. J. Magnes. Alloys.

[4] Elaiyarasan U., Satheeshkumar V., Senthilkumar C. (2019). Microstructure study on electro discharge deposited magnesium alloy with semi sintered and sintered electrode. Mater. Res. Express.

[5] Reza Kashyzadeh K., Amiri N., Maleki E., Unal O. (2023). A Critical Review on Improving the Fatigue Life and Corrosion Properties of Magnesium Alloys via the Technique of Adding Different Elements. J. Mar. Sci. Eng..

[6] Yang Y.-Y., Chen H.-S., Zhou J., Nie H.-H., Xu X., Xi S.-X., Chang Y.-L. (2023). Study on interface behavior and mechanical properties of Al/Cu laminated tubes fabricated by strong staggered spinning at room temperature. J. Mater. Res. Technol..

[7] Li L., Chen S., Lu Q., Shu X., Zhang J., Shen W. (2023). Effect of Process Parameters on Spinning Force and Forming Quality of Deep Cylinder Parts in Multi-Pass Spinning Process. Metals.

[8] Lexian H., Dariani B.M. (2009). Effect of roller nose radius and release angle on the forming quality of a hot-spinning process using a non-linear finite element shell analysis. Proc. Inst. Mech. Eng. Part B J. Eng. Manuf..

[9] Mohebbi M., Akbarzadeh A. (2010). Experimental study and FEM analysis of redundant strains in flow forming of tubes. J. Mater. Process. Technol..

[10] Essa K., Hartley P. (2010). Optimization of conventional spinning process parameters by means of numerical simulation and statistical analysis. Proc. Inst. Mech. Eng. Part B J. Eng. Manuf..

[11] Liu Y., Shu X., Cen Z., Li Z., Ye B. (2021). Effects of Process Parameters on Surface Straightness of Variable-Section Conical Parts during Hot Power Spinning. Appl. Sci..

[12] Xia Y., Shu X., Zhu Y., Li Z. (2020). Influence of Process Parameters on Forming Load of Variable-Section Thin-Walled Conical Parts in Spinning. Appl. Sci..

[13] Wang Y., Zhang X. (2020). Experimental study on the residual stress of the power spinning rod bushing with main technological parameters. Mater. Today Proc..

[14] Mohebbi M.S., Rahimi Pour M. (2019). Effects of temperature, initial conditions, and roller path on hot spinnability of AZ31 alloy. Int. J. Adv. Manuf. Technol..

[15] Jahazi M., Ebrahimi G. (2000). The influence of flow-forming parameters and microstructure on the quality of a D6ac steel. J. Mater. Process. Technol..

[16] Molladavoudi H.R., Djavanroodi F. (2011). Experimental study of thickness reduction effects on mechanical properties and spinning accuracy of aluminum 7075-O, during flow forming. Int. J. Adv. Manuf. Technol..

[17] Keneshlou M., Biglari F.R., Shafaie M. (2023). A numerical and experimental analysis of noncircular blank spinning. J. Manuf. Process..

[18] Xia Q., Long J., Xiao G., Yuan S., Qin Y. (2021). Deformation mechanism of ZK61 magnesium alloy cylindrical parts with longitudinal inner ribs during hot backward flow forming. J. Mater. Process. Technol..

[19] Abd-Eltwab A.A., El-Abden S., Ahmed K.I., El-Sheikh M., Abdel-Magied R.K. (2017). An investigation into forming internally-spline sleeves by ball spinning. Int. J. Mech. Sci..

[20] Watson M., Long H., Lu B. (2015). Investigation of wrinkling failure mechanics in metal spinning by Box-Behnken design of experiments using finite element method. Int. J. Adv. Manuf. Technol..

[21] Su J., Sanjari M., Kabir A.S.H., Jung I.-H., Yue S. (2016). Dynamic recrystallization mechanisms during high speed rolling of Mg–3Al–1Zn alloy sheets. Scr. Mater..

[22] Wang J., Kumar M.A., Beyerlein I.J. (2023). Investigation of crossed-twin structure formation in magnesium and magnesium alloys. J. Alloys Compd..

[23] Wang H., Liao Z., Cai W. (2022). Prediction on springback angle and process paramater optimization in electro-assisted spinning for AZ31B magnesium alloy. Forg. Stamp. Technol..

[24] Xie X., Zhang W., Yang T., Cao W. (2016). Spinning of Ti55531 titanium alloy cylinder. Aerosp. Mater. Process..

[25] Quigley E., Monaghan J. (2000). Metal forming: An analysis of spinning processes. J. Mater. Process. Technol..

[26] Kim N., Kim H., Jin K. (2012). Optimal Design to Reduce the Maximum Load in Ring Rolling Process. Int. J. Precis. Eng. Manuf..

[27] Prasad Y., Rao K. (2009). Effect of homogenization on the hot deformation behavior of cast AZ31 magnesium alloy. Mater. Des..

[28] Özel T. (2006). The influence of friction models on finite element simulations of machining. Int. J. Mach. Tools Manuf..

[29] Zhao X., Mu Z., Zhao H., Wang P., Song W., Yang G. (2023). Influence of Inner Roller Geometric Parameters on Counter-Roller Spinning with 6061 Aluminum Alloy Tube. Metals.

[30] Cao Z. (2015). Study on the Power Spinning Process of AZ80 Magnesium Alloy. Bachelor’s Thesis.

[31] Panda A., Sahoo A.K., Rout A.K. (2016). Multi-attribute decision making parametric optimization and modeling in hard turning using ceramic insert through greys relational analysis: A case study. Decis. Sci. Lett..

[32] Gürcan S., Serkan A. (2023). Taguchi and Gray Relational Analysis Optimization of Cutting Parameters during Face Milling of Cryogenic Treated Aluminum 6061 Alloys Using Cryogenic and Non-cryogenic Inserts. J. Mater. Eng. Perform..

[33] Juliyana S.J., Prakash J.U., Rubi C.S., Salunkhe S., Gawade S.R., Nasr E.S.A., Kamrani A.K. (2023). Optimization of Wire EDM Process Parameters for Machining Hybrid Composites Using Grey Relational Analysis. Crystals.

[34] Jangra K.K., Sharma N., Khanna R., Matta D. (2016). An experimental investigation and optimization of friction stir welding process for AA6082 T6 (cryogenic treated and untreated) using an integrated approach of Taguchi, grey relational analysis and entropy method. Proc. Inst. Mech. Eng. Part L J. Mater. Des. Appl..

[35] Agapovichev A.V., Khaimovich A.I., Smelov V.G., Kokareva V.V., Zemlyakov E.V., Babkin K.D., Kovchik A.Y. (2023). Multiresponse Optimization of Selective Laser Melting Parameters for the Ni-Cr-Al-Ti-Based Superalloy Using Gray Relational Analysis. Materials.

